# Investigating Internalization of Reporter-Protein-Functionalized Polyhedrin Particles by Brain Immune Cells

**DOI:** 10.3390/ma17102330

**Published:** 2024-05-14

**Authors:** Krishma A. K. Parwana, Priyapreet Kaur Gill, Runyararo Njanike, Humphrey H. P. Yiu, Chris F. Adams, Divya Maitreyi Chari, Stuart Iain Jenkins

**Affiliations:** 1 School of Life Sciences, Keele University, Keele ST5 5BG, UK; x3w98@students.keele.ac.uk (K.A.K.P.); c.adams@keele.ac.uk (C.F.A.); 2 School of Medicine, Keele University, Keele ST5 5BG, UK; x5b11@students.keele.ac.uk (P.K.G.); x3q49@students.keele.ac.uk (R.N.); 3 School of Engineering & Physical Sciences, University of Edinburgh, Edinburgh EH14 4AS, UK; h.h.yiu@hw.ac.uk; 4 Neural Tissue Engineering Keele (NTEK), Keele University, Keele ST5 5BG, UK

**Keywords:** microglia, nanoparticles, microparticles, polyhedra, GFP, neural, crystals, drug delivery, drug depot, nucleus

## Abstract

Achieving sustained drug delivery to the central nervous system (CNS) is a major challenge for neurological injury and disease, and various delivery vehicles are being developed to achieve this. Self-assembling polyhedrin crystals (POlyhedrin Delivery System; PODS) are being exploited for the delivery of therapeutic protein cargo, with demonstrated efficacy in vivo. However, to establish the utility of PODS for neural applications, their handling by neural immune cells (microglia) must be documented, as these cells process and degrade many biomaterials, often preventing therapeutic efficacy. Here, primary mouse cortical microglia were cultured with a GFP-functionalized PODS for 24 h. Cell counts, cell morphology and Iba1 expression were all unaltered in treated cultures, indicating a lack of acute toxicity or microglial activation. Microglia exhibited internalisation of the PODS, with both cytosolic and perinuclear localisation. No evidence of adverse effects on cellular morphology was observed. Overall, 20–40% of microglia exhibited uptake of the PODS, but extracellular/non-internalised PODS were routinely present after 24 h, suggesting that extracellular drug delivery may persist for at least 24 h.

## 1. Introduction

Sustained delivery of therapeutic molecules to the CNS is a major challenge, limiting current therapies for neurological injury and disease. Development of drug delivery vehicles is a major area of research, with drives to identify biocompatible structures suitable for facilitating transit across the blood–brain barrier (BBB), evading immune clearance, possible cell-specific targeting and with therapeutically appropriate drug release profiles [1,2].

Proteins such as growth factors have substantial potential as pro-repair drugs, for example, for stimulating nerve fibre outgrowth and angiogenesis with the recruitment of progenitor/stem cells [3,4,5], but achieving sustained delivery within the CNS is challenging [6]. In particular, physiological conditions (including temperature, immune cell clearance and degradative enzymes) reduce the biological activity of most growth factors, resulting in a short half-life [~0.5–1.7 h for nerve growth factor (NGF)] [7,8] which is likely insufficient for therapeutic efficacy.

Injected/implanted drug depots or delivery vehicles can be used to slowly release drugs such as growth factors, maintaining more consistent pharmacological effects over weeks or months [9,10,11], e.g., implanted mechanical pumps, degradable scaffolds including hydrogels and transplanted encapsulated cells secreting neurotrophic factors [12,13,14].

Polyhedrin protein matrices (polyhedra) have emerged as a novel drug delivery system [15]. They have been used to form drug delivery vehicles known as PODS (POlyhedrin Delivery Systems) which can encapsulate therapeutic molecules, with gradual release of this cargo over days/weeks [13,16]. Polyhedrin is encoded by genes in baculoviruses and cypoviruses, which use this protein to generate paracrystalline matrices (occlusion bodies; 0.15–3 µm in size [16,17]) protecting viruses against dehydration, UV radiation, freezing, acidic conditions and dissolution [18] for several years [19,20,21]. Polyhedrin self-assembles into cuboid structures within infected insect cells, and this process has been exploited for bioengineering of drug delivery PODS. By genetically engineering insect cells to express high levels of polyhedrin, the subsequent self-assembling polyhedra can incorporate other proteins, as ‘cargo’, in place of the virus.

For experimental and therapeutic applications, genes for cargo proteins are modified to include a polyhedrin-binding peptide sequence (immobilisation tag; e.g., N-terminal 50 amino acids of virus turret protein, or polyhedrin H1-helix) and then co-expressed with polyhedrin within in vitro cell cultures [22]. This immobilisation tag ensures incorporation of the cargo within the co-crystals [23]. The same promoter sequence is used for polyhedrin and the cargo protein, ensuring comparable quantities of each. Reported sizes of PODS vary from 200 to 5000 nm, with 500–2000 nm being typical [24].

To achieve drug delivery, PODS are reportedly degraded, releasing encapsulated cargo proteins. Drug release can be tailored to produce physiologically relevant concentrations: EC_50_ of 6–300 pmol/L for NGF and brain-derived neurotrophic factor (BDNF) [6,25]. The precise mechanisms by which PODS and associated cargo are processed by mammalian cells are not clearly understood as naturally occurring polyhedra are resistant to pH levels as low as 2, suggesting resistance to lysosomal conditions, and resistant to alkaline conditions below pH 10.5 (polyhedra dissolve at pH 10.5 in the insect larval midgut, releasing viral particles) [22]. When implanted into bone, PODS remained present at 10 weeks but not 15 weeks, suggesting complete degradation in the absence of highly alkaline conditions [26]. Cargo release from PODS at pH <10.5 has been suggested to involve a range of proteases.

Matrix metalloproteinase-2 (MMP-2; a collagenase) and MMP-8 (a gelatinase) showed degradation of green fluorescent protein-functionalised PODS (GFP-PODS) during culture with the PC12 neuron-like cell line [27]. Trypsin, chymotrypsin, MMP-3 and MMP-7 may also degrade PODS, but these enzymes are known to degrade the GFP signal used as a proxy for PODS degradation, confounding the interpretation of the findings.

As MMP-2 and -8 are present in connective tissue, including within the CNS [28], PODS can be expected to degrade and release cargo during most therapeutic applications. Although protease degradation is required for cargo release, the cargo itself would need to be resistant to these enzymes, such that the drugs remain bioavailable [27]. Growth factors have evolved to function within extracellular matrix (ECM) in the presence of MMPs; therefore, growth factor cargoes may be expected to persist despite the presence of MMPs in vivo [27].

PODS have been tested for drug delivery in various cell types and in vivo systems (Table 1). Supernatant from otic neuronal progenitor cultures, analysed by ELISA, showed release of brain-derived neurotrophic factor (BDNF) from PODS at a rate of 25–50 pg/mL/day, cumulatively releasing 300 pg/mL over 7 d [13]. In contrast, a dose of 20,000 pg/mL BDNF (without a PODS or other vehicle) was reduced to only 100 pg/mL after 3 d, highlighting the benefits of bioencapsulation for prolonging bioavailability. Despite these benefits, PODS have received little attention for neural applications.

When considering CNS drug delivery using any biomaterial, it is critical to document interactions with the brain immune cells: microglia [30,31]. Microglial roles include surveillance for tissue damage and pathogens which are cleared and digested, but this property can limit the efficacy of ‘foreign’ biomaterials [32]. Microglia are a substantial extracellular barrier to nanoparticle (NP) delivery, showing avid levels of NP uptake and outcompeting other neural cell types in co-cultures [30,33]. Any subsequent death of microglia, presumably due to degraded NP materials intracellularly, could become a cause of secondary pathology [34].

Accordingly, the efficacy of PODS for drug delivery within the CNS will depend critically on whether the PODS are endocytosed by microglia and whether subsequent toxic or inflammatory responses are induced in these cells.

Uptake of PODS by CNS microglia has not been investigated, although they have been tested with peripheral macrophages and shown to be internalised without adverse effects [24]. For example, PODS did not impair macrophage mobility, chemotaxis or migration (relevant to tumour infiltration). The authors also speculated that the resistant properties of PODS enabled cargo to survive processing within endosomes, including lysosomes [24].

The goal of this study was to investigate the interactions of reporter-protein-functionalised polyhedrin crystals with primary brain microglia. Our objectives were three-fold:

(1) To establish the extent to which PODS are internalised by microglia;
(2) To assess whether acute toxic effects or microglial activation are induced by PODS internalisation;
(3) To document intracellular localisation of PODS.

## 2. Materials and Methods

The care and use of animals was in accordance with the Animals (Scientific Procedures) Act of 1986 (United Kingdom) with approval by Keele University’s School of Life Sciences Ethics Committee (SLEC). Keele University Establishment licence number: X350251A8.

### 2.1. Materials

Unless otherwise stated, tissue culture-grade plastics, media and media supplements were from Fisher Scientific (Loughborough, UK) and Sigma-Aldrich (Poole, UK). DAPI mounting medium was from Vector Laboratories (Peterborough, UK). Secondary antibodies were from Jackson ImmunoResearch Laboratories Inc. (West Grove, PA, USA).

Fluorescent PODS (loaded with green fluorescent protein, GFP; excitation, 488 nm; emission, 510 nm) were from Cell Guidance Systems (Cambridge, UK). The manufacturer reports the GFP-PODS as being isolated from a *Spodoptera frugiperda* (Sf9) cell culture and then lyophilised. The polyhedrin sequence expressed in these cells is from *Bombyx mori* cypovirus. The polyhedrin and GFP sizes were reported by the manufacturer as 28.7 and 32.1 kDa respectively, and the sequences contain 250 and 284 amino acids, respectively [35].

GFP-PODS were reconstituted in double-distilled H_2_O as per the manufacturer’s instructions (200 × 10^6^ PODS/mL) and stored at 4 °C. Presumably based on BMP-2 as the cargo, Matsumoto et al. estimated that 36 × 10^6^ PODS likely contain ~1 µg of cargo protein (0.067 pg/POD) based on equivalent biological activity rather than direct measures of protein [26]. This may be expected to vary substantially for larger or smaller cargo proteins and will obviously differ across PODS of different sizes.

### 2.2. Characterisation of PODS Particles

Micrographs of PODS particles were taken using phase contrast and fluorescence microscopy and both scanning and transmission electron microscopy (SEM and TEM). For SEM, particles were coated using layered OsO_4_ and thiocarbohydrazide (OTOTO protocol [36]) and then imaged using a Hitachi S4500 FESEM (5 kV accelerating voltage). For TEM, cells cultured on aclar were fixed (2.5% glutaraldehyde in 0.1 M sodium cacodylate, 2 nM CaCl_2_; 2 h), then postfixed (1% OsO_4_; 1 h), dehydrated (ethanol, 70%, 90%, 100%, 100% dry; 15 min each) and embedded (3:1 Spurr’s resin:100% dry ethanol overnight at room temperature (RT), followed by pure Spurr’s resin, four times). Finally, samples were baked in moulds with fresh resin (16 h, 600 °C). Eighty-nanometer sections were dried on copper grids (Reichert-Jung Ultracut-E ultramicrotome; Buffalo, NY, USA), stained (uranyl acetate, 20 min; lead citrate, 5 min) and imaged (FEI Techni G2; Thermo Fisher scientific, Waltham, MA, USA).

### 2.3. Primary Mouse Microglia Culture

Primary mixed glial cultures were prepared from dissociated cerebral cortices of SD1 mice at postnatal days 1−3; then, high purity microglial populations were isolated by sequential rotary shaking procedures using well-established protocols [37]. Microglia were seeded (5 × 10^4^ cells cm^−2^) on poly-D-lysine (PDL)-coated glass coverslips in 24-well plates and maintained in D10 medium [Dulbecco’s modified Eagle medium (DMEM) supplemented with 10% foetal bovine serum (FBS), 2 mM glutaMAX-I, 1 mM sodium pyruvate, 50 U mL^−1^ penicillin and 50 μg mL^−1^ streptomycin] at 37 °C in 5% CO_2_/95% humidified air [38].

### 2.4. Incubation of PODS with Microglia

After allowing microglia to adhere overnight, the GFP-PODS were heavily vortexed and then diluted in fresh D10 media at 4 × 10^5^ PODS mL^−1^. A 100% medium change was performed, with PODS being vortexed immediately prior to addition to ensure good particle dispersion (500 µL total volume; 200,000 PODS/well; ~2.1 PODS/cell, based on initial seeding density; 24 h incubation time).

### 2.5. Fixation and Immunostaining of Cultures

Cultures were washed with phosphate-buffered saline (PBS) and then fixed using 4% paraformaldehyde in PBS (20 min; room temperature, RT). Fixed cells were incubated with a blocker (5% normal donkey serum in PBS, 0.3% Triton X-100 at RT; 30 min) and then primary antibody in blocker (goat anti-Iba1, abcam ab5076; 1:1000; 4 °C; overnight). Cells were then washed (PBS), incubated with blocker (RT; 30 min) and incubated with Cy3-conjugated secondary antibody in blocker (1:200; RT; 2 h). Finally, coverslips were washed (PBS) and mounted with the nuclear stain DAPI.

### 2.6. Microscopy and Image Analysis

The size of the PODS was determined by measuring two perpendicular side lengths per crystal in merged fluorescence-phase contrast micrographs; 59 PODS were measured (×100 objective; ImageJ software, NIH) [39].

Immunostained samples were imaged using an Axio Scope A1 fluorescence microscope and counterpart micrographs were merged using Zen software (Zeiss, Oberkochen, Germany). Z-stack imaging was performed to confirm that PODS were intracellular (Axio Observer.Z1, Zeiss, Oberkochen, Germany).

A minimum of three microscopic fields per culture were assessed for all conditions. Individual cells were manually delineated and then measured in red and green channels, providing morphometric data and integrated density values for GFP and Iba1 staining (ImageJ software, NIH) [39]. Corrected total cell fluorescence (CTCF) was used for analyses of Iba1 intensity, with the background subtracted (average of five cell- and PODS-free regions: blanks) [38].

CTCF = cell integrated density − (cell area × mean pixel intensity of blank regions)

Feret’s (maximum) diameter represents the furthest two points of each cell (caliper diameter). Feret’s minimum diameter indicates the shortest distance at which two parallel lines can restrict the entire cell. The aspect ratio was determined by dividing Feret’s maximum by Feret’s minimum. For a circle, this value is 1; therefore, more amoeboid morphologies will be closer to 1. Solidity (area/convex hull area) also represents circles as 1, with more undulating/ramified shapes decreasing from 1.

### 2.7. Statistical Analyses

Each culture was established from a separate mouse litter (*n* = 4 biological replicates). Data were analysed using Prism statistical analysis software (GraphPad, Boston, MA, USA) and are expressed as mean ± standard error of the mean (SEM) unless declared otherwise.

## 3. Results

### 3.1. Characterisation of PODS Particles

**Figure 1:**
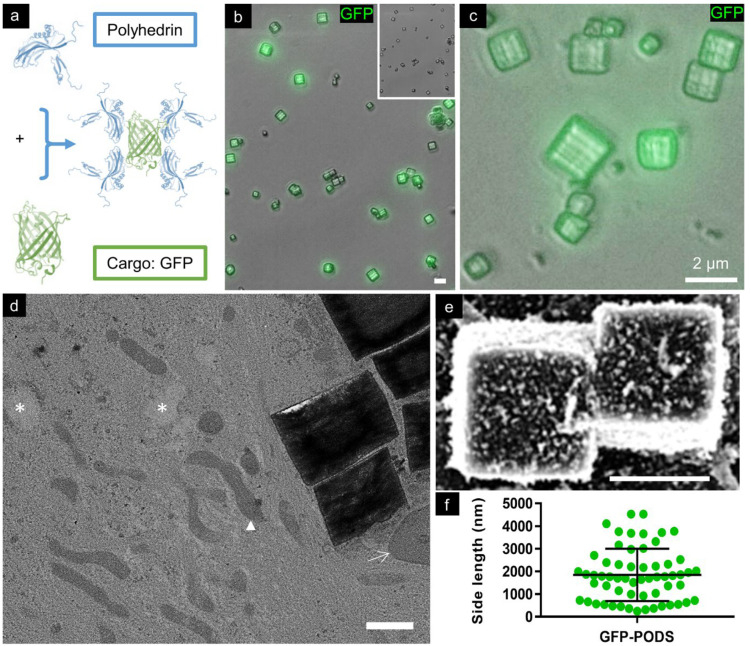
PODS are co-crystals of polyhedrin and a cargo protein—in this instance GFP—and were readily imaged by microscopy. (**a**) Schematic indicating co-crystalline lattice structure of GFP-PODS. (**b**) Merged phase contrast and fluorescence micrograph illustrating consistent morphologies of GFP-PODS and limited aggregation (inset shows phase contrast micrograph alone). (**c**) Merged phase contrast and fluorescence micrograph illustrating size range of GFP-PODS and their tendency to ‘stand’ flush to a flat surface, presenting a squared upper surface. (**d**) Transmission electron micrograph of intracellular PODS. PODS were found in the cytosol, amongst the organelles. Arrow shows a tangential section of a cell nucleus, arrowhead points to a mitochondrion and * indicates vacuole-like structures. Scale bar: 1 μm. (**e**) Scanning electron micrograph of GFP-PODS, showing cuboid morphology; scale bar: 2 μm. (**f**) Graph of PODS side length; error bars indicate standard deviation.

GFP-PODS were readily visualised by phase contrast and fluorescence microscopy as well as scanning and transmission electron microscopy (Figure 1). The mean side length of the GFP-PODS was 1.85 ± 1.14 µm (standard deviation; range, 0.25–4.52 µm), consistent with previous descriptions. The longer edge was typically within 12% of the other (perpendicular) measured edge, and right-angled corners were evident. Consistent with the manufacturer’s report that PODS are denser than water and adhere to most culture plastics, particles were observed drifting to the base of the culture plates within minutes of addition and typically appeared to be flush with the surface, presenting a square upper facet, as per the expected cuboid structure.

### 3.2. No Acute Toxicity Was Evident in Microglia Incubated with PODS Particles

**Figure 2:**
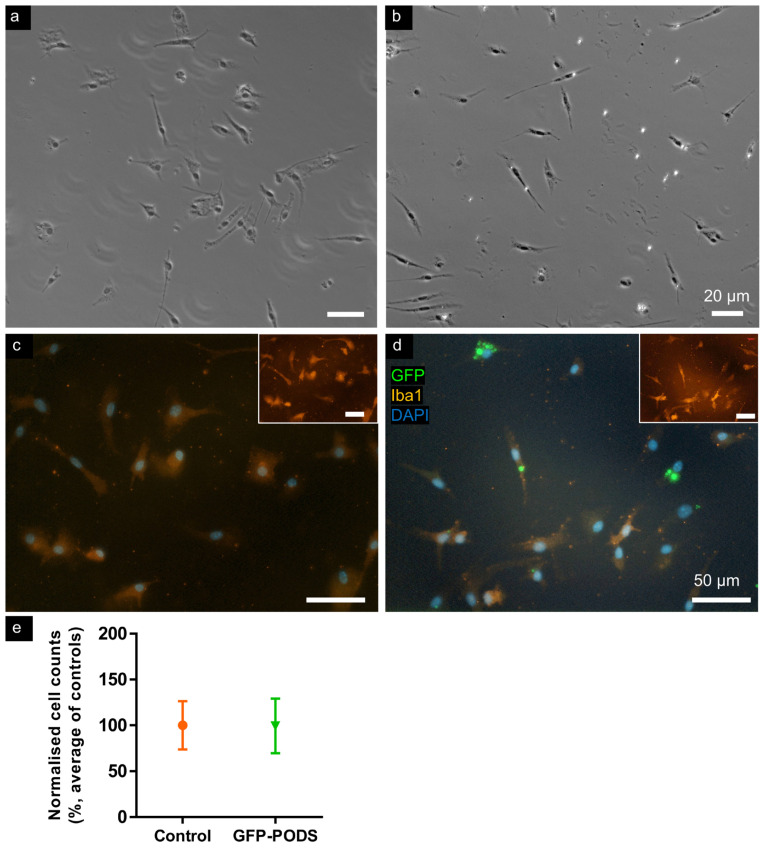
Microglia incubated with GFP-PODS did not exhibit acute toxicity. Similar cell numbers and morphologies were observed in control and GFP-PODS-treated microglial cultures. Low magnification phase contrast micrographs of (**a**) control and (**b**) PODS-treated microglial cultures. Higher magnification merged fluorescence micrographs of (**c**) control and (**d**) PODS-treated microglial cultures (insets show Iba1 staining alone). (**e**) Graph indicating similar cell counts in control and GFP-PODS-treated cultures. Data normalised to average of all control counts; no significant difference (*p* = 0.838), two-tailed unpaired *t*-test, *n* = 4.

Microglial cultures treated with GFP-PODS resembled control (untreated) cultures (Figure 2). Microglia were identified by Iba1 immunostaining and exhibited similar ranges of morphologies—mostly ramified, with some amoeboid—in each condition. Cultures were >98% Iba1^+^. Cell counts were comparable between treated and control cultures, and no apoptotic or pyknotic cells were observed.

### 3.3. Morphometry of Microglia Incubated with PODS Was Unchanged versus Controls

**Figure 3:**
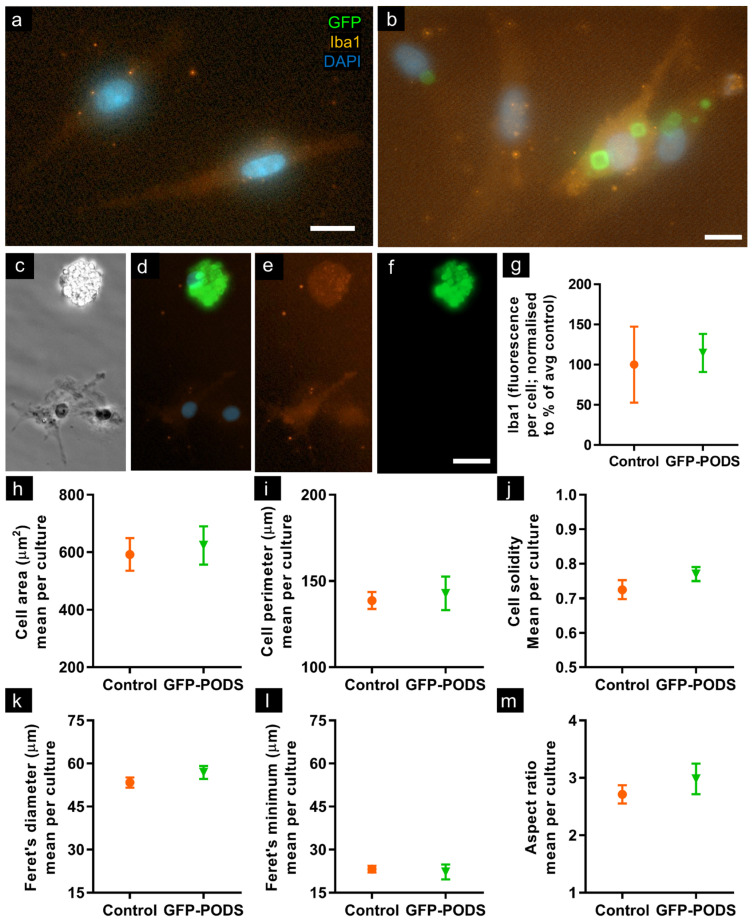
Detailed morphological analyses found no differences between control and PODS-treated cultures, and Iba1 expression was similar. (**a**) Merged fluorescence micrograph of control culture showing lack of green fluorescence and ramified Iba1+ microglia. (**b**) Merged fluorescence micrograph of Iba1+ microglia in PODS-treated culture. Note intracellular GFP-PODS distributed throughout the cytosol. Cell morphologies are similar to those in control cultures, with similar quantities and dimensions of processes. Occasional instances of extensive PODS uptake were observed, as shown in (**c**–**f**): counterpart phase contrast, merged fluorescence, red channel fluorescence and green channel fluorescence micrographs, respectively. Note amoeboid morphology. (**g**) Graph showing similar intensity of Iba1 expression in control and PODS-treated microglial cultures. Graphs comparing various cellular morphometrics: (**h**) area, (**i**) perimeter, (**j**) Feret’s (max) diameter, (**k**) Feret’s min diameter, (**l**) Feret’s aspect ratio and (**m**) solidity. All graphs show no significant differences from two-tailed unpaired *t*-tests, *n* = 4; *p*-values: (**g**) 0.285, (**h**) 0.801, (**i**) 0.292, (**j**) 0.648, (**k**) 0.718, (**l**) 0.226 and (**m**) 0.429. Scale bars: 10 µm.

Iba1 expression was not noticeably greater in cells exhibiting PODS uptake, and no significant difference in intensity was determined between control and GFP-PODS-treated cultures. The following morphological measures of microglia were compared between treated and control cultures (Figure 3): cellular area, perimeter, solidity, Feret’s diameter, Feret’s minimum diameter and aspect ratio. No significant differences were noted for any of these parameters.

### 3.4. Perinuclear PODS Occasionally Distorted Cell Nuclei

**Figure 4:**
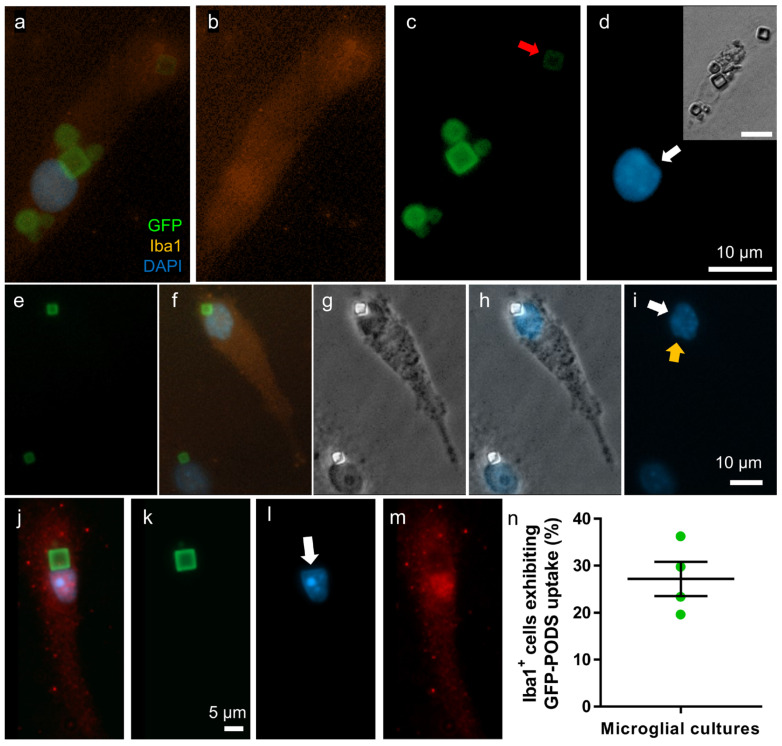
PODS showed both cytosolic and perinuclear localisation and occasionally distorted the shape of the nucleus. (**a**–**d**) Micrographs showing Iba1+ microglia with intracellular GFP-PODS, both perinuclear and cytosolic, including particle distant to nucleus (red arrow): (**a**) merged fluorescence, (**b**) red channel only, (**c**) green channel only and (**d**) blue channel only, with phase contrast inset. White arrow indicates flattened edge of nucleus, which coincides with flat face of PODS particle. (**e**–**i**) Micrographs of Iba1+ microglia, each with a perinuclear PODS particle: green, merged, phase contrast, phase contrast-blue merge and blue. White arrow indicates angled indent in nucleus edge, coinciding with corner of particle. Orange arrow indicates similar dimple in region without PODS particle. Such observations left doubt as to whether PODS were genuinely displacing the nuclear envelope. (**j**–**m**) Fluorescence micrographs of Iba1+ microglial cell: merged, green, blue and red. White arrow indicates flattened edge of nucleus, co-localised with flat edge of PODS particle. Supplementary Materials: video file showing z stack microscopy of this cell and particle. (**n**) Graph indicating percentage of Iba1+ microglial cells that exhibited PODS uptake within four separate cultures. Error bars indicate SEM.

Intracellular PODS particles were observed in perinuclear locations and more distantly within the cytosol (Figure 4). Perinuclear particles were occasionally observed seemingly distorting the nuclear membrane, coincident with a straight edge or corner of a PODS particle. Microscopy using a ×100 objective and a z-stack microscope was used to confirm these observations (see Supplementary Materials for video). Some extracellular particles were observed in all cultures, but the majority of particles were internalised by microglia, with 20–40% of microglia in each culture having at least one intracellular PODS particle.

## 4. Discussion

GFP-PODS were characterised in isolation and then incubated with primary neural immune cells, microglia, to assess cell responses and the fate of the PODS. The PODS were consistent in morphology, being recognisably cuboid, and both extracellular and intracellular particles retained these shapes during 24 h of exposure to cells. Green fluorescence (due to GFP cargo) was obvious and persisted during the 24-h timeframe of the experiment (for both extracellular and intracellular PODS), showing these particles to be compatible with a range of fluorescence microscopy and facilitating the tracking of PODS.

Across cultures, ca. 20–40% of microglia were found to contain an intracellular PODS, with intracellularity confirmed using z-stack microscopy.

Extracellular GFP-PODS were regularly observed after 24 h incubation with microglia, which is in contrast to other NPs tested by our group, which showed dramatic levels of internalisation even at 1 h post-incubation [30,40]. Few of these particles remained extracellular after 24 h exposure. This implies that some as yet undefined property of the PODS renders them less susceptible to immune cell uptake. This could be related to particle shape [41] or chemistry and would require further investigation. Additionally, no evidence of particle degradation was observed for GFP-PODS here over a period of 24 h exposure. The ultimate fate of intracellular PODS, including possible degradation or exocytosis, requires further study in more prolonged experiments. However, our data do suggest that this limited immune cell uptake and degradation could make PODS an attractive vehicle for use as a CNS drug depot, without confounding effects of avid immune clearance.

We observed several cells having nuclei distorted by an immediately neighbouring PODS particle (Figure 4). To our knowledge, such an effect has not been reported for PODS or other particles. Whether there are any consequences for cellular function is unclear; however, no obvious aberrations in cellular morphology were observed, such as loss of adherence, pyknosis or cell shrinkage, which would be indicative of cell damage.

Assessment of microglial morphologies and cell counts did not reveal evidence of acute toxicity within the cell populations analysed, consistent with reports for other cell types (Table 1), although few reports have presented detailed cellular level analyses. No obvious pro-inflammatory features were observed within this study, such as increased prevalence of amoeboid morphologies or increased Iba1 expression. This is consistent with the peripheral macrophage study [24], although detailed assays of cytokine release or oxidative stress would be warranted for firm conclusions regarding inflammatory responses.

It is not clear whether the observed microglial internalisation of PODS will be a barrier to CNS drug delivery. PODS are specifically synthesised with protein cargo, and the majority of proteinaceous therapeutics are identical or analogous to endogenously produced proteins, including growth factors. If PODS are degraded within microglia over a longer timeframe, releasing a protein cargo that is endogenously produced by microglia such as BDNF, then it may be anticipated that these molecules will be recognised and processed identically to endogenously produced molecules.

Peripheral macrophages and microglia are sources of various pro-repair factors. It may be predicted that such cells may internalise drug-functionalised PODS and become living drug delivery depots. It would also be necessary to assess whether drug processing/release is dependent upon that specific protein being expressed within macrophages, or whether protein cargoes not expressed in microglia/macrophages may also be secreted by these cells.

Some studies have suggested that particle shape may influence cellular endocytosis and intracellular processing [41]. It might be hypothesised that some particle shapes give rise to beneficial outcomes in terms of trafficking to the nucleus or trafficking that avoids a lysosomal fate. Some PODS particles may be small enough for micropinocytotic uptake (typically <0.3 μm) [42]; however, given the size range of PODS, it seems likely that the majority of particles are too large for uptake mechanisms other than phagocytosis (>20 μm capacity [43]) and macropinocytosis (~5 μm [44]). We are unaware of any studies assessing specific endocytotic mechanisms of PODS uptake, although some reports suggest uptake may be via ‘phagocytosis’ [24,25]. Given that different uptake mechanisms may lead to different intracellular fates, typically lysosomal for larger entities [42,45,46], it will be important to determine which specific mechanisms each cell type uses for PODS uptake.

**Figure 5:**
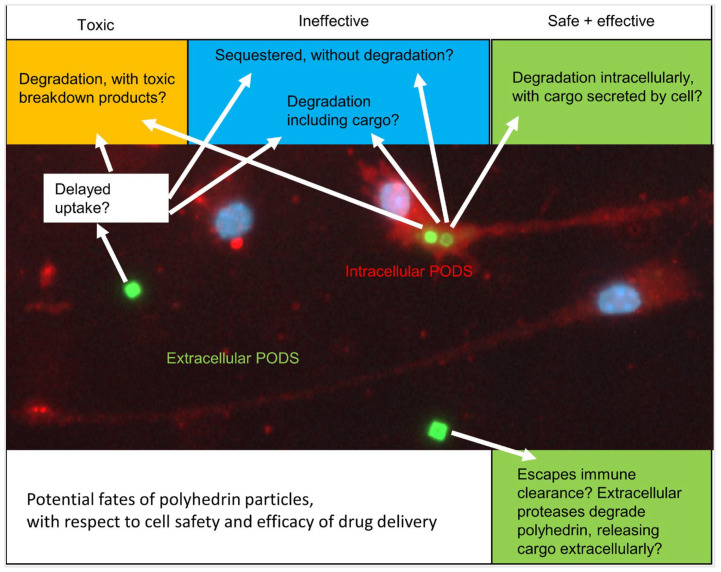
Possible fates for intra- and extracellular PODS particles. This schematic offers speculation on possible PODS fate, depending on whether PODS are internalised by cells, and then whether there is degradation of the polyhedrin and whether intracellular release of cargo would result in secretion of the cargo into extracellular space, or whether the cargo may be sequestered or also subject to intracellular degradation. Prolonged resistance to immune cell uptake (extracellular PODS) would be beneficial for extracellular drug release, as would cellular uptake followed by drug secretion (green background). However, microglial sequestration of PODS without cargo release, or with degradation of cargo, would prevent drug delivery (blue background). Finally, the worst-case scenario would be cellular clearance followed by degradation, resulting in toxic breakdown products leading to cytotoxicity, possibly to the extent of cell death (orange background).

Several scenarios may be proposed for the ultimate fate of PODS. For all PODS that persist extracellularly, effective drug release can be expected. However, for internalised PODS particles, multiple possible fates are possible. PODS may be internalised and then sequestered without degradation (stasis), or cargo may be degraded along with the polyhedrin, in both cases preventing drug delivery. Internalisation may alternatively result in degradation with cytotoxic breakdown products, impairing cell function or even resulting in cell death. However, if PODS are degraded intracellularly, with cargo release and subsequent processing of cargo leading to extracellular secretion, then effective drug delivery may still be achieved even for internalised PODS (Figure 5).

The various particle fates proposed in Figure 5 are speculative and warrant longer-term studies lasting weeks or months and involving co-cultures of multiple neural cell types. Here, we have documented acute PODS–cell interactions, including toxicity and possible microglial activation.

For particles that remain extracellular, it can be anticipated that proteases will eventually degrade them entirely, with released cargo either inducing biological responses or also being degraded. For biomaterials/particles that are internalised, their fate is at least partly dependent on which uptake mechanisms first introduce that particle into the cell interior [47,48]. This has long been considered true for particles based on their size, with possible size ranges having been documented for the various endocytotic mechanisms (some mechanisms having a maximum possible size for their cargo) [42]. Notably, PODS have been used with cargo of up to 109 kDa in size (432 amino acids; BMP-7) [25]. Further investigation will be required to establish whether particle shape (and other physicochemical features) influences uptake methods and subsequent intracellular processing.

## 5. Conclusions

Microscopic analyses, including z-stack microscopy and TEM, led us to conclude that primary derived microglia are capable of internalising polyhedrin co-crystals, PODS, with some internalised PODS observed to distort the shape of the cell nucleus, an observation not previously reported. However, such distortions were not found to be associated with obvious toxic or inflammatory cell responses, and analyses at the level of whole cultures also indicated no reduction in cell number or increase in inflammatory morphologies. Some PODS remained extracellular during 24 h experiments, supporting the conclusion that extracellular drug release from PODS is likely to occur within brain tissue, even in the presence of microglia.

## Figures and Tables

**Table 1:** Publications testing the POlyhedrin Delivery System (PODS) with neural cells or peripheral immune cells.

Cell Type, Source (Author) [Ref]	PODS Type/Cargo	PODS Concentration	Exposure Time (Days)	Uptake	Toxicity	Cell Responses to PODS (Other Notes)
Rat PC12 cell line (Matsuzaki) [27]	NGF, EGFP	5 or 10 × 10^4^ PODS/coverslip (7 × 10^4^ cells)	5 (no media change)	Not assessed	“no evidence of inflammatory or foreign-body reaction” in vivo (PODS in collagen scaffold, implanted into bone)	Did not address direct cell responses to PODS. Cells avoided dried patch of PODS, applied before cell seeding. Drug delivery seemed effective. (Reported protease-induced pores in PODS.)
hESC-derived otic neuronal progenitors (Chang) [13]	hBDNF	80 × 10^4^ PODS/well	7	Not assessed	Not directly assessed; no toxicity noted	Did not address direct cell responses to PODS Drug delivery seemed effective (consistent hBDNF release over 7 d, assessed by ELISA)
THP1-derived macrophages, M0, M1, M2 (Wendler) [24]	empty, EGFP, IL-6, FGF2, −10	5, 10 or 15 PODS/cell	1, 4	Yes	15 PODS/cell did not show toxicity, up to 96 h; but “apoptotic bodies” at ~50 PODS/cell	All phenotypes (M0, M1, M2) showed uptake; ‘almost all PODS at 24 h’. No effects on cellular function reported. IL-6 secretion by M1 was unaltered by empty-/FGF10-PODS uptake (lack of inflammation). IL-6-PODS: IL-6 in media from cell culture was 8–30% of IL-6-PODS alone.
Mouse primary bone marrow monocytes (Wendler) [24]	M-CSF, GM-CSF	5 or 10 PODS/cell	1	Yes	”seemingly without negatively influencing their behavior”	Cargo protein remained bioactive after macrophage uptake; possibly secreted by macrophages? PODS suggested to survive acidic conditions of phagolysosomes.
Human chondrocytes; ‘non-professional phagocytes’ (Whitty) [25]	empty, BMP-2, -7	50 ng/mL, 25–200 ng/mL	14	Yes	No	Uptake reported as “phagocytosis”, although endocytotic mechanism not specifically assessed. Extent of uptake only assessed as ‘efficient’. (Increased proliferation when treated with PODS-BMP2 and -BMP7)
Spinal ganglion cells, from hiPSCs (Nella) [29]	hBDNF	2 or 80 × 10^4^ PODS/well	7	Not assessed	Not assessed	Drug delivery/release produced cellular responses. (Serum was required to release BDNF from PODS in cell-free conditions; Authors speculate that proteases are necessary for cargo release.)

BMP2, 7: bone morphogenetic protein 2, 7; EGFP: Enhanced Green Fluorescent Protein; FGF-2,-10: Fibroblast Growth Factor 2, 10; GM-CSF: Granulocyte-Macrophage Colony-Stimulating Factor; hBDNF: human brain derived neurotrophic factor; hESC: human embryonic stem cell; hiPSC: human induced pluripotent stem cells; IL-6: interleukin-6, inflammatory cytokine; M0/M1/M2: unactivated/inflammatory/anti-inflammatory macrophage phenotype; M-CSF: Macrophage Colony-Stimulating Factor; NGF: Nerve Growth Factor; PC12: rat pheochromocytoma cell line; PODS: POlyhedrin Delivery System; THP-1: human monocyte cell line.

## Data Availability

The raw data supporting the conclusions of this article will be made available by the authors on request.

## Supplementary Materials

Supplementary data can be downloaded at: https://www.mdpi.com/article/10.3390/ma17102330/s1: ###. Video S1: GFP-PODS_uglia_zStack.avi. This shows z-stack microscopy, descending through an Iba1^+^ microglial cell containing a GFP-PODS particle clearly abutting the nucleus and inducing a flattened nuclear edge. Excerpts from this video are shown in Figure 4.
